# Employing template-directed CRISPR-based editing of the *OsALS* gene to create herbicide tolerance in Basmati rice

**DOI:** 10.1093/aobpla/plac059

**Published:** 2023-01-13

**Authors:** Kashaf Zafar, Muhammad Zuhaib Khan, Imran Amin, Zahid Mukhtar, Mehak Zafar, Shahid Mansoor

**Affiliations:** Agricultural Biotechnology Division, National Institute for Biotechnology and Genetic Engineering (NIBGE), Constituent College of Pakistan Institute of Engineering and Applied Sciences, Jhang Road, Faisalabad 37000, Pakistan; Department of Biotechnology, Balochistan University of Information Technology, Engineering and Management Sciences (BUITEMS), Quetta 87300, Pakistan; Agricultural Biotechnology Division, National Institute for Biotechnology and Genetic Engineering (NIBGE), Constituent College of Pakistan Institute of Engineering and Applied Sciences, Jhang Road, Faisalabad 37000, Pakistan; Agricultural Biotechnology Division, National Institute for Biotechnology and Genetic Engineering (NIBGE), Constituent College of Pakistan Institute of Engineering and Applied Sciences, Jhang Road, Faisalabad 37000, Pakistan; Agricultural Biotechnology Division, National Institute for Biotechnology and Genetic Engineering (NIBGE), Constituent College of Pakistan Institute of Engineering and Applied Sciences, Jhang Road, Faisalabad 37000, Pakistan; Agricultural Biotechnology Division, National Institute for Biotechnology and Genetic Engineering (NIBGE), Constituent College of Pakistan Institute of Engineering and Applied Sciences, Jhang Road, Faisalabad 37000, Pakistan; Agricultural Biotechnology Division, National Institute for Biotechnology and Genetic Engineering (NIBGE), Constituent College of Pakistan Institute of Engineering and Applied Sciences, Jhang Road, Faisalabad 37000, Pakistan

**Keywords:** Acetolactate Synthase (ALS), Basmati, bispyribac-sodium (BS), CRISPR/Cas9, herbicide, homology-directed repair (HDR), rice

## Abstract

Rice (*Oryza sativa*) is one of the primary food crops which contributes major portion of daily calorie intake. It is used as model crop for various genome editing studies. Basmati rice was also explored for establishing non-homologous end joining-based genome editing. But it was not clear whether homology-directed repair (HDR)-based genome editing can be done in Basmati rice. The current study was designed to establish HDR-based genome editing in Basmati rice to develop herbicide tolerance. There is severe weed spread when rice is grown via direct planted rice method in various countries to save labour and water resources. Therefore, the use of herbicides is necessary to control weeds. These herbicides can also affect cultivated rice which creates the need to develop herbicide-tolerant rice. In current study, we introduced a point mutation in *Acetolactate Synthase* gene to convert tryptophan to leucine at position 548. For this purpose, different constructs for HDR were tested with different RNA scaffold and orientation of repair templates. Out of four different architectures, the one having repair template identical to the target DNA strand precisely edited the target site. We successfully established template-directed CRISPR-Cas9 system in Super Basmati rice by detecting desired substitutions at the target site in *Acetolactate Synthase* locus. Moreover, this editing of *Acetolactate Synthase* gene resulted in the production of herbicide tolerance in Super Basmati rice. This study suggests that such type of HDR system can be used to precisely edit other genes for crop improvement.

## Teaser

Rice is an important food crop, also used as model crop for studying genome editing. Basmati rice was also explored for establishing genome editing by our group, but template-based genome editing was not done in this rice variety. The aim of the current study was to develop herbicide tolerance in Basmati rice. This was done by introducing a point mutation in the *Acetolactate Synthase*gene to convert tryptophan to leucine at position 548. For this purpose, different constructs for homology-directed repair were tested and one of them precisely edited the target site. In this way template-directed CRISPR-Cas9 system was successfully established in Super Basmati rice by detecting desired substitutions, resulting in the production of herbicide tolerance.

## Introduction

Rice (*Oryza sativa*) is the second most frequently cultivated crop and is consumed as a primary food in various parts of the world (Sasaki 2001; Pazuki and Sohani 2013). It serves more than 90 % of Asian population by contributing 50–80 % of daily calories (Khush 2005; Zeigler and Barclay 2008). Amongst different rice cultivars, Basmati rice is well-known for having long grain, fragrance and flavour. Every year, demand for rice production is increasing because of increased consumption rate of 1.6–1.8 % (Shobarani *et al.* 2010). To fulfil this increasing demand, the production of rice should be increased. Rice cultivation requires large amount of water which is becoming a key hindrance in its production. According to an estimate, about 3/4th of the world and Asian water resources are consumed for cultivation of rice (Bouman *et al.* 2007). The continuous decline in surface and groundwater table has become a foremost limiting factor in rice cultivation (Farooq *et al.* 2009). The conventional method of growing rice is very demanding and requires intensive labour input and a large amount of water (Bhushan *et al.* 2007; Bouman *et al.* 2007). To reduce labour input and water resources, direct planted rice (DPR) in aerobic environment provides an eye-catching alternative to the farmers in many countries (Farooq *et al.* 2009). However, the major concern in adopting this method is severe weed spread which limits its wider acceptance (Rao *et al.* 2007). In comparison with traditionally planted rice (TPR), this widespread presence of weeds in DPR is very difficult to control (Tomita *et al.* 2003; Rao *et al.* 2007). This severe weed infestation causes delays in growth and loss in grain quality and yield. In order to control these weeds, different herbicides are applied in the field. Alongside weeds, these herbicides also affect cultivated rice. To cope this challenge, a promising strategy will be to develop herbicide tolerance in rice (Shoba *et al.* 2017; Piao *et al.* 2018; Wang *et al.* 2019; Han *et al.* 2020).

Among different herbicides, bispyribac-sodium (BS) and chlorsulfuron are used to deal with a wide spectrum of grasses and weeds. However, the major concern is that it can also target acetolactate synthase (*ALS*) gene of rice. *ALS* is involved in a pathway which synthesizes branched-chain amino acids (isoleucine, leucine and valine) (Chipman *et al.* 1998; McCourt and Duggleby 2006; Liu *et al.* 2016). In order to be effective only against weeds, modification of *ALS* gene without affecting the phenotype is necessary. A mutant of *OsALS* gene (*OsALSR*) was identified by prior studies using tissue culturing which was able to tolerate pyrimidinyl carboxy herbicide (BS) (Osakabe *et al.* 2005; Kawai *et al.* 2007). Out of different reported point mutations, two were crucial for BS resistance. These are G to T substitution which changes amino acid tryptophan into leucine at position 548 (W548L) and another G to T which modify serine at position 627 into isoleucine (S627I) (Shimizu *et al.* 2005). Although, a single substitution at 548 to modify tryptophan to leucine (W548L) is enough to create herbicide tolerance in rice crop (Endo *et al.* 2007).

Genome editing technology holds a great importance for crop improvement because it can precisely modify DNA sequences (Zafar *et al.* 2021, 2022). Clustered Regularly Interspaced Short Palindromic Repeats and CRISPR-associated Cas9 nuclease (CRISPR-Cas9) is simple, versatile and easily customizable tool due to which it had been used in past for trait improvement of different crops (Cong *et al.* 2013; Alam *et al.* 2022; Liu *et al.* 2022). There are many reports of using this tool in rice crop by exploiting both repairing methods non-homologous end joining (NHEJ) and homology-directed repair (HDR) (Zafar *et al.* 2020b). The frequency of targeted integration by HDR is lower than that of NHEJ (Puchta 1998; Li *et al.* 2019, 2020). That’s why most of the editing events reported for modifying plant’s genome were done via error-prone NHEJ (Miao *et al.* 2013; Ma *et al.* 2015). Hence, establishing a technique that can introduce specific mutations at the desired position will greatly help in crop improvement (Weeks *et al.* 2016). Exploiting cell’s HDR pathway after CRISPR-Cas9 generated double-stranded break (DSB) at the specific location can potentially provide a robust approach to achieve precise modification at the desired locus. In many eukaryotes including plants, HDR-mediated improvements through CRISPR-Cas9 have been reported previously (Doudna and Charpentier 2014; Butt *et al.* 2017). This approach replaces chromosomal segments or genes with the inserted DNA by genetic recombination between the target chromosomal segment and the introduced DNA (Gloor *et al.* 1991; Donoho *et al.* 1998; Bibikova *et al.* 2003; Fauser *et al.* 2012). The HDR is relatively challenging in plants than NHEJ because the frequency of the targeted integration by HDR remains very low (Ray and Langer 2002; Puchta 2004; Song and Stieger 2017; Wang *et al.* 2017; Miki *et al.* 2018). The Nipponbare rice has been modified for developing herbicide tolerance using HDR in the past (Sun *et al.* 2016; Butt *et al.* 2017). But the Basmati rice was not exploited for this purpose. In this study, we have achieved HDR events in Super Basmati rice. Previously, we have mutated *SWEET14* promoter region exploiting NHEJ pathway in Super Basmati rice for bacterial blight resistance (Zafar *et al.* 2020a). Here we established HDR and tested a strategy (Butt *et al.* 2017) to introduce point mutations (W548L) in the *OsALS* gene of Super Basmati rice using CRISPR-Cas9-mediated homologous recombination to develop resistance against BS herbicide.

## Materials and Methods

### Plant material

The Indica rice variety, Super Basmati (a native Pakistani variety of rice), was used to achieve CRISPR-Cas9-mediated HDR by incorporating two point mutations. The rice seeds were collected from DNA markers and the Applied Genomics Lab of National Institute for Biotechnology and Genetic Engineering, Faisalabad, Pakistan. The seeds were dehusked and healthy seeds were chosen for sterilization using ethanol (70 %). The seeds were then soaked for 20 min in 50 % (v/v) commercial bleach solution with continuous stirring. After washing seeds four to five times with sterile water, they were placed on MS medium (Murashige and Skoog 1962) containing 2,4-dichlorophenoxyacetic acid and vitamins (callus induction media) for formation of callus.

### BS sensitivity measurement in callus and seedlings

BS sensitivity was measured for rice callus and seedlings. The 3–4 weeks old growing calli were subcultured on fresh callus induction medium supplemented with 0, 0.1, 0.2, 0.3 and 0.4 μM BS. Callus growth was visually monitored and compared after 25 days. For seed germination, dehusked and disinfected seeds were placed in sterile water at 30^o^C for 2 days. The seeds were then placed in sterile water having 0, 0.25, 0.5, 1.0, 1.5, 2.0, 2.5, 3.0, 4.0, 5.0 μM BS under 16-h light/8-h dark photoperiod at 30 °C. The seeds were observed continuously for root and seedling development and data were recorded after 5 days.

### Template designing and construct development

**Figure 1.**
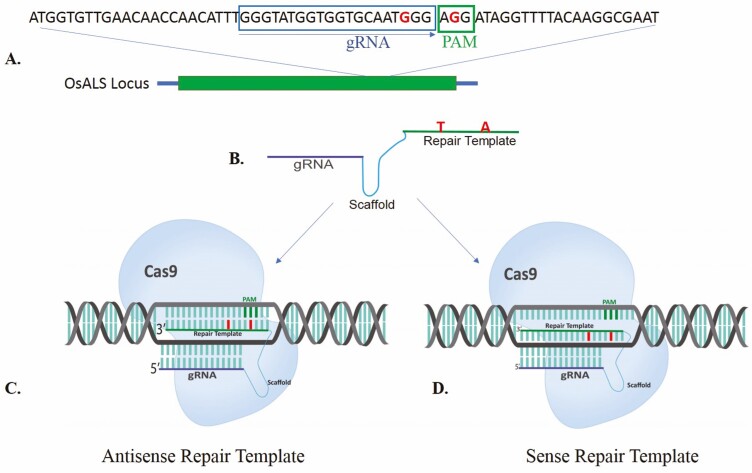
Schematic of targeting *OsALS* locus using different architectures. (A) *OsALS* locus is shown at the top. Sequence of the target region is shown by indicating PAM sequence and gRNA (B) Architecture of the construct having gRNA, scaffold RNA and repair template with required changes (C) Antisense repair template is shown relative to gRNA, where repair template is complementary to the non-target strand. (D) Sense repair complementary to the target strand. In both sense and anti-sense templates, gRNA, scaffold RNA and repair template are shown with required changes.

**Figure 2.**
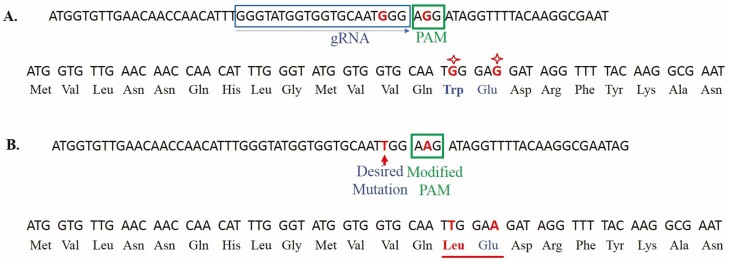
DNA and protein sequence of the target strand before and after editing. (A) Sequence of the wild type locus indicating gRNA and PAM sequences. The bases which will be substituted are marked. Below is the triplet codon and amino acid sequence. (B) Sequence of modified strand is shown. Substitution of G to T in gRNA region changed tryptophan to leucine and G to A in PAM region does not change amino acid (area underlined). The triplet codons and amino acid sequence of the modified strand is shown below.

**Figure 3.**
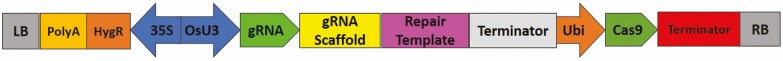
Schematic figure of construct used for template-directed editing. General schematic presentation of the constructs. Based on gRNA scaffold and orientation of repair template, multiple constructs were developed. The gRNAs were expressed under *OsU3* promoter and codon-optimized Cas9 was expressed under *Ubiquitin* promoter.

The target region was amplified using Super Basmati rice genomic DNA to confirm the exact sequence before designing guide RNA (gRNA). The genomic DNA was extracted using cetyl trimethylammonium bromide method (Stewart 1993) and the target region was amplified using primer set *OsALS*-F and *OsALS*-R (Table 1). Amplified fragment of 496 base pair (bp) was cloned and sequenced (Thermo Fisher Scientific, USA; pTZ57 R/T vector). To incorporate G to T substitution at the target site, gRNA (5ʹ GGGTATGGTGGTGCAATGGG**AGG** 3ʹ) was designed to alter amino acid at position 548. The underlined AGG represents the PAM sequence. A repair template was also designed to mutate the targeted sequence. This repair template had two mutations, one mutation was introduced to convert tryptophan to leucine (T**G**G to T**T**G) at position 548 (Fig. 1) and the second point mutation was inserted next to leucine codon to modify PAM sequence. This was a synonymous substitution (GA**G** to GA**A**) and both encode glutamate and therefore did not change the amino acid (Fig. 2). The complete synthesized fragment consisting of gRNA, scaffold RNA, repair template (with desired substitutions) and terminator was cloned into pRGEB32 vector (Addgene plasmid # 63142) (Xie *et al.* 2015) under *OsU3* promoter. Four different types of fragments, having differences in scaffold RNA and orientation of repair template, were used (Table 2). The scaffold RNAs are very important in CRISPR-mediated genome editing because they provide a modular system for transcriptional programming at a specific locus. The scaffold encodes information about which locus to target and also delivers instructions about regulatory functions to be performed at that locus (Zalatan *et al.* 2015). Different scaffold RNAs were reported in the past, i.e. yeast scaffold RNA which has been used for rice (Xie *et al.* 2015), and previously described human scaffold RNA (Chen *et al.* 2013; Zalatan *et al.* 2015). Both scaffold RNA sequences were tested to determine if there were any differences between yeast (Xie *et al.* 2015; Butt *et al.* 2017) and human scaffold RNA sequences (Chen *et al.* 2013; Zalatan *et al.* 2015; Butt *et al.* 2017) to perform HDR-mediated genome editing in Basmati rice. The repair template was used in the sense and antisense orientations with respect to gRNA sequence. The sense template was complementary to the strand which was targeted by gRNA, whereas antisense template was complementary to non-target strand (Fig. 1C and D). The pRGEB32 vector has rice codon-optimized Cas9 expressed under *OsUbiquitin* promoter (the complete cassette is shown in Fig. 3). The cassette was initially confirmed by PCR and later by Sanger sequencing.

**Table 1.** Primers used in the study

Sr. no.	Purpose	Primer set	Product length (bp)
1	To amplify target region	*OsALS*-F: ATGGTAGCTTCCTCATGAACA	496
		*OsALS*-R: GAGCACATACAAACATCATAGG	
2	To amplify vector fragment	Vec-F: GGTGCTACCAGCAAATGCTGGAAGCCG	427*
		Vec-R: CCCGAATTTGTGGACCTGCAGGCATGC	620**

*Product length was 427 when amplifying simple vector.

**Product length was approximately 620 when using vector with cassette.

**Table 2.** Sequences of the four different modalities for template-directed CRISPR-Cas9 editing

Sr. no.	gRNA/repair template	5ʹ-Sequence-3ʹ
1	OsALS-g-Antisense	
	Line 1: BsaI restriction site	^**1**^Ggtctctggca
	Line 2: gRNA sequence	^**2**^GGGTATGGTGGTGCAATGGG
	Line 3: Yeast gRNA scaffold	^**3**^GTTTTAGAGCTAGAAATAGCAAGTTAAAATAAGGCTAGTCCG TTATCAACTTGAAAAAGTGGCACCGAGTCGGTGC
	Line 4: Antisense repair template	^**4**^TTCCGGGTTGCCCAAGTATGTATGCGCCCTATTCGCCTTGTAA AACCTATC**t**TCC**a**ATTGCACCACCATACCCAAATGTTGGTTGTT CAACACCATCACC
	Line 5: Terminator	^**5**^TTTTTTTTTT
	Line 6: BsaI restriction site	^**6**^gttttgagacc
2	OsALS-h-AntiSense	
	Line 1: BsaI restriction site	^**1**^Ggtctctggca
	Line 2: gRNA sequence	^**2**^GGGTATGGTGGTGCAATGGG
	Line 3: Human scaffold	^**3**^GTTTAAGAGCTATGCTGGAAACAGCATAGCAAGTTTAAATAA GGCTAGTCCGTTATCAACTTGAAAAAGTGGCACCGAGTCGGTGC
	Line 4: Antisense repair template	^**4**^TTCCGGGTTGCCCAAGTATGTATGCGCCCTATTCGCCTTGTAAAACCTATC**t**TCC**a**ATTGCACCACCATACCCAAATGTTGGTTGTT CAACACCATCACC
	Line 5: Terminator	^**5**^TTTTTTTTTT
	Line 6: BsaI restriction site	^**6**^gttttgagacc
3	OsALS-g-Sense	
	Line 1: BsaI restriction site	^**1**^Ggtctctggca
	Line 2: gRNA sequence	^**2**^GGGTATGGTGGTGCAATGGG
	Line 3: Yeast gRNA scaffold	^**3**^GTTTTAGAGCTAGAAATAGCAAGTTAAAATAAGGCTAGTCCGTTATCAACTTGAAAAAGTGGCACCGAGTCGGTGC
	Line 4: Sense repair template	^**4**^GGTGATGGTGTTGAACAACCAACATTTGGGTATGGTGGTGCAAT**t**GGA**a**GATAGGTTTTACAAGGCGAATAGGGCGCATACATACTTGGGCAACCCGGAA
	Line 5: Terminator	^**5**^TTTTTTTTTT
	Line 6: BsaI restriction site	^**6**^gttttgagacc
4	OsALS-h-Sense	
	Line 1: BsaI restriction site	^**1**^Ggtctctggca
	Line 2: gRNA sequence	^**2**^GGGTATGGTGGTGCAATGGG
	Line 3: Human scaffold	^**3**^GTTTAAGAGCTATGCTGGAAACAGCATAGCAAGTTTAAATAAGGCTAGTCCGTTATCAACTTGAAAAAGTGGCACCGAGTCGGTGC
	Line 4: Antisense repair template	^**4**^GGTGATGGTGTTGAACAACCAACATTTGGGTATGGTGGTGCAAT**t**GGA**a**GATAGGTTTTACAAGGCGAATAGGGCGCATACATACTTGGGCAACCCGGAA
	Line 5: Terminator	^**5**^TTTTTTTTTT
	Line 6: BsaI restriction site	^**6**^gttttgagacc

The bold small letters in repair template are representing the modified bases.

### Rice transformation

Embryogenic calli (3–4 weeks old) were used for *Agrobacterium*-mediated transformation as described by Hiei and Komari (2008) with MS media (Murashige and Skoog 1962) as a basal medium. Four constructs were developed to target the desired location with same gRNA. Approximately, 1000 calli were transformed for each construct. The herbicide-resistant calli were selected on 0.8 μM BS (32967 Sigma-Aldrich). After completing 2–3 weeks of selection on 0.8 μM BS medium, alive and proliferating calli were placed initially on BS-supplemented pre-regeneration and then on regeneration media. The calli showing regeneration were shifted for rooting in sterile jars having half-strength MS media. The seedlings were kept on rooting medium till the development of roots. Plants were then shifted to soil-containing pots and were grown at 28 °C in greenhouse till maturity.

### Sequencing

The gRNA and Cas9 cassette were confirmed by primer set 3 (Vec-F and Vec-R) (sequence is given in Table 1). Amplification of approximately 620 bp confirmed the presence of binary construct. The Cas9-targeted region of *OsALS* gene was amplified from all the plants using primer set 1 (sequence given in Table 1). The PCR products were purified, cloned into vector pTZ57 R/T (Thermo Fisher Scientific, USA) and then sequenced using Sanger sequencing.

### Herbicide resistance and phenotyping

The edited seeds were observed for germination. Few seeds from both, wild-type and edited, plants were grown in water and observed visually. To confirm herbicide resistance, seeds were dehusked, sterilized and soaked in water. The germinated seeds were placed in water, supplemented with 15 μM BS. The seeds were monitored continuously, and data were recorded after 7 days. The root and shoot development were also compared after 15 days. The 120 μM BS was then sprayed on plants and phenotype was observed after 40 days.

## Results

### BS sensitivity in callus and seedlings

**Figure 4.**
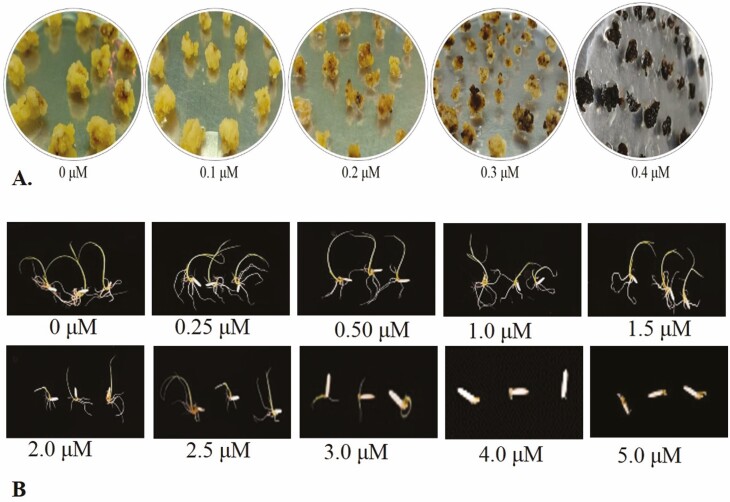
BS sensitivity of Super Basmati rice callus and seedlings. (A) BS sensitivity of callus on BS-supplemented medium at 0, 0.1, 0.2, 0.3 and 0.5 μM, respectively. The degree of callus growth was compared after 25 days after. (B) Seedling growth on different BS concentrations from 0 to 5.0 μM.

This experiment was conducted to get information which was essential for the selection of edited and herbicide-resistant-edited plants of Super Basmati. To check BS sensitivity of Super Basmati seedlings, fresh seeds were grown. After 2 days, seeds were transferred to various concentrations of BS and growth inhibition was recorded in terms of minor or no (++), slight (+), moderate (±), severe (x) and complete (--) growth inhibition (inhibition scale at the bottom of Table 3). Accordingly, like untreated control, the seedling growth was not inhibited at 0.25 μM. Then, the concentration was increased from 0.5 to 1.5 μM and a slight growth inhibition was observed. From 2.0 to 2.5 μM concertation, there was moderate inhibition. At 3.0 μM concentration, severe growth inhibition was recorded. When the concentration was increased further (4.0–5.0 μM), complete growth inhibition was observed (Fig. 4B; Tables 3 and 4). These results indicate that increasing BS concentration above 4 μM completely inhibited the growth of Super Basmati seedlings.

**Table 3.** BS sensitivity of Basmati seeds

BS concentration (μM)	Number of seeds	Number of seeds showing signs of growth inhibition	Percentage of growth inhibition	Seedling growth inhibition
0	50	0	0 %	++
0.25	50	1	2 %	++
0.5	50	4	8 %	+
1.0	50	5	10 %	+
1.5	50	7	14 %	+
2.0	50	12	24 %	±
2.5	50	15	30 %	±
3.0	50	26	52 %	X
4.0	50	48	96 %	--
5.0	50	49	98 %	--
Growth inhibition scale				
++	(0–5 %)	Minor or no		
+	(6–15 %)	Slight		
±	(≥20)	Moderate		
X	(≥50 %)	Severe		
--	(≥95 %)	Complete		

**Table 4.** BS sensitivity of Basmati calli

BS concentration (μM)	Number of calli	Number of calli showing signs of growth inhibition	Calli growth inhibition
0	100	2	++
0.1	100	3	++
0.2	100	20	±
0.3	100	51	X
0.4	100	99	--

The growth of callus grown on various BS concentrations was visually examined and compared with untreated control (Fig. 4A). After transferring to BS-supplemented media, till Day 6, there was evenly vigorous growth of callus on all BS concentrations (0, 0.1, 0.2, 0.3 and 0.4 μM). After Day 6, growth inhibition in callus was observed. After spending 25 days on BS-supplemented medium, growth data were recorded and compared. Like untreated control, growth inhibition was not observed at 0.1 μM concentration; a moderate inhibition was observed at 0.2 μM concentration. When the concentration was increased to 0.3 μM, severe growth inhibition was observed. In comparison to seedlings, the callus showed higher sensitivity to BS, because the callus growth was completely inhibited at 0.4 μM (Fig. 4A; Tables 3 and 4).

### Template architecture for genome editing

To investigate template-directed CRISPR-based editing in indigenous Super Basmati rice, we have designed different modalities to understand which could work effectively for this variety. This method was selected to test in Basmati rice on the basis of its success in Nipponbare rice (Butt *et al.* 2017). The RNA template-mediated repairing was chosen for establishing HDR. The HDR using RNA as template was testified in human cells and yeast (Derr *et al.* 1991; Storici *et al.* 2007; Nowacki *et al.* 2008; Keskin *et al.* 2014). It was reported that RNA–DNA hybrids are more stable than DNA–DNA duplexes. The RNA transcripts can also be produced in higher amounts in the cell providing more templates for successful HDR. That’s why in the current study RNA template system was selected (Chein and Davidson 1978; Li *et al.* 2019). The RNA repair template was designed in two different orientations (Fig. 1). The first template was used in the antisense orientation, where the repair template was homologous to target strand (Fig. 1C). The second template was in the sense orientation, having complementarity to the target DNA strand (Fig. 1D). Two scaffold sequences, yeast scaffold and human scaffold, with the sense and antisense orientation to create four different modalities were used (Chen *et al.* 2013; Xie *et al.* 2015; Zalatan *et al.* 2015) (Table 2). These four designs targeted the desired sequence. The targeting of repair templates at the desired location was facilitated by Cas9 endonuclease. Cas9 has performed two functions, created DSBs at the targeted position and as loader to provide repair template at the site of DSB. In this way we use a bifunctional molecule to perform editing through HDR at the desired locus. However, the re-editing by CRISPR-Cas9 was prevented by introducing a synonymous mutation in repair template which distorted the PAM sequence. As described below, out of these four architectures, only a single model, having yeast scaffold RNA and with antisense orientation, gave the best results. We were unable to get any results with human scaffold RNA and template with sense orientation.

### Template-mediated editing to confer herbicide resistance

To assess whether template-mediated targeted genome editing can work for Super Basmati, we carefully chosen *OsALS* gene to introduce DSB via CRISPR-Cas9 and template-directed fixing of this DSB to develop herbicide (BS)-resistant rice. The *OsALS* is a very important enzyme which is required for the synthesis of branched-chain amino acids valine, leucine and isoleucine (Chipman *et al.* 1998). Various structurally different classes of herbicides (imidazolinones, sulfonylureas, triazolopyrimidine sulfonamides and pyrimidinyl carboxy herbicides) target *OsALS* (Shimizu *et al.* 2005). Among these structurally distinct groups, BS is from group ‘pyrimidinyl carboxy herbicide’.

**Figure 5.**
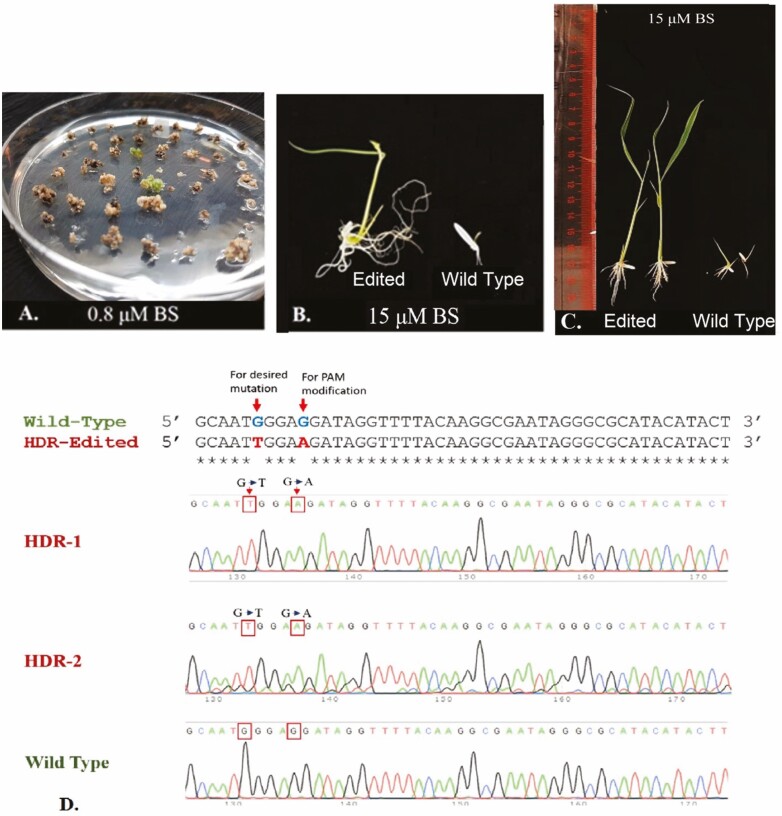
Template directed editing to confer herbicide resistance. (A) Survival of edited callus on 0.8 μM BS supplemented media. The proliferating callus indicated with arrows are showing herbicide resistance. (B) Seedling growth of edited and wild type seeds on 15 μM BS. (C) Root and shoot development of wild type and edited seeds shown after 15 days of being at 15 μM BS. (D) Sanger sequencing of edited lines highlighting substituted bases in boxes.

We tested various template architectures and gRNAs platform to generate BS resistance in Super Basmati. To transform rice, *Agrobacterium*-mediated transformation method was used and MS medium (supplemented with 0.5 and 0.8 μM BS) was chosen to select proliferating callus with herbicide resistance (Fig. 5A). The edited callus cells were able to survive and regenerate on BS-supplemented media. The plantlets were further evaluated by PCR and Sanger sequencing (Fig. 5D). Out of 2000 transformed calli, we were only able to recover eight plantlets after selection process. All recovered plantlets were from same repairing template architecture (antisense template with yeast scaffold RNA sequence). The sequencing results revealed that out of these eight plantlets five were observed with desired substitutions which were introduced using template-directed HDR. We were able to get HDR in Super Basmati with 0.5 % efficiency (Table 5). We were unable to get any regeneration with other modalities. The herbicide resistance was further confirmed by screening of the seeds. For this purpose, germinated seeds were allowed to grow on 15 μM BS and a clear difference was observed in the wild-type and edited seeds. Seeds from all the progenies were tested for possible herbicide tolerance and the seeds from two progenies were able to grow normally on herbicide (Fig. 5B). The root and shoot development of wild-type and edited seeds was also compared after 15 days (Fig. 5C). The BS was then sprayed on plants; the wild-type plants died after 40 days, whereas edited plants grew normally. The sequencing of the target region of these plants also confirmed that resistance was achieved due to substitution at the targeted site (Fig. 5D), thus signifying the applicability of this HDR-mediated platform for targeted genome editing in Super Basmati rice.

**Table 5.** Editing efficiency of CRISPR-Cas9 in Basmati rice using HDR

gRNA/repair template	BS conc	No. of transformed calli	Survived on selection	Plants produced	No. of plants recovered	No. of edited plants	Editing efficiency (%)*
OsALS-g-Antisense	0.5	1000	50	5	3	0	0
	0.8	1000	100	6	5	5	0.5

No regeneration was observed with other modalities (OsALS-h-AntiSense, OsALS-g-Sense, OsALS-h-Sense).

*(No. of edited plants/No. of transformed calli) × 100.

## Discussion

The current applications of CRISPR-Cas9-mediated HDR-based targeted improvements in numerous eukaryotes, including plants, have increased our capacity to engineer targeted genomic locations (Doudna and Charpentier 2014; Butt *et al.* 2017). For wide-ranging applications and targeted improvement of crops, substantial progress is required in CRISPR-Cas9-mediated genome editing via HDR (Doudna and Charpentier 2014; Hsu *et al.* 2014; Verma and Greenberg 2016). Multiple approaches have been exploited previously to increase HDR efficiency like increasing gRNA and Cas9 concentration, inhibiting NHEJ using small molecules, choosing a precise cell-cycle stage to perform editing (Bi *et al.* 2014; Lin *et al.* 2014; Wang *et al.* 2015; Weber *et al.* 2015; Van Chu *et al.* 2018). Rice crop has been used for performing HDR-mediated genome editing, but Super Basmati rice was not exploited for this purpose. In this study, the Cas9 endonuclease was used to perform dual functions, to create DSBs at the target site and for delivering the repair template at the site of DSB to perform precise DNA repair. The *OsALS* gene was used for this purpose to create resistance against BS herbicide.

Herbicide (BS) sensitivity was measured for callus and seedings of Super Basmati rice. Comparatively, calli were more sensitive to BS than seedlings because they were only able to tolerate 0.3 μM concentration of BS (Fig. 4A). However, this concentration was higher than other previously tested Indica rice varieties. For instance, Kasalath (KL) was able to tolerate maximum concentration of 0.1 μM BS (Taniguchi *et al.* 2010). The seedlings were able to tolerate up to 3.0 μM concentration of BS. Further increase in BS concentration to 4 μM completely inhibited the seedling growth (Fig. 4B). As Super Basmati rice belongs to Indica group, so the results were consistent with the previous studies which showed that Indica rice seedling were less sensitive to BS than Japonica rice seedlings (Ohno *et al.* 2008; Taniguchi *et al.* 2010). However, Super Basmati seedlings showed less sensitivity than previously tested Indica rice varieties, where maximum seedling growth was observed at 2.5 μM BS and increasing the concentration beyond 2.5 μM completely inhibited the seedling growth (Taniguchi *et al.* 2010).

After measuring BS sensitivity, *OsALS* gene of Super Basmati rice was targeted to introduce two point mutations at the target site in order to engineer resistance against BS. We have designed constructs having gRNA and repair templates for creation and repair of DSB via cellular HDR pathway. Different architectures were tried to find the most suitable architecture for Super Basmati rice. Among them, one architecture was able to perform Cas9-mediated DSBs creation at target site and subsequent repairing using donor template. This architecture contains rice scaffold RNA with repair template in antisense orientation. Our results vary from the previously reported study by Butt *et al.* (2017) in which they got desired results with human scaffold RNA. However, repair template was in the antisense orientation (Butt *et al.* 2017).

In this study, the length of the repair template was 100 bp. These repair templates were designed to repair *OsALS* gene via HDR after Cas9-mediated DSBs. It carried two substitutions which has to be incorporated at the target locus. The applicability of this system was confirmed by successful editing of *OsALS* gene in Super Basmati genome to engineer resistance against BS. The *OsALS* gene was targeted previously via HDR-mediated CRISPR-Cas9-based genome editing of *ALS* by testing various architectures in Nipponbare rice but the applicability of this system in Indica rice especially in Basmati background was not known before (Butt *et al.* 2017). There are many possibilities of using various substrates for DNA repair including RNA, DNA (single- or double-stranded) and RNA–DNA hybrids. Numerous probable mechanisms could be proposed for this type of targeted editing. In this study, RNA repair template was used. Constructs with various architectures produced considerably different results. For example, all constructs having repair template with a sequence complementary to the target strand were unable to produce any editing events at the target site. Similarly, none of the constructs having human scaffold RNA mediate any targeted editing, suggesting that crop variety also matters while performing genome editing. Similarly, it has been observed that same constructs can behave in different manner in distinctive rice varieties.

## Conclusion

In conclusion, we exploited a system in Super Basmati rice which worked as a guide for DSB formation and its subsequent repair using RNA template. We established the applicability of this template-directed CRISPR-Cas9 system by successfully detecting substitutions at the target site in *OsALS* locus (Fig. 5D). This editing of *OsALS* gene resulted in the production of herbicide tolerance in Super Basmati. The herbicide-tolerant rice can be cultivated via DPR to save labour and water resources. Moreover, this template-directed DNA repair system can be used to precisely edit other genes for trait improvement of crops. Although, the efficiency of this system was low in Super Basmati rice. Recently, an attempt was made where Cas9–VirD2 fusion was used to increase efficiency of HDR in Nipponbare (Ali *et al.* 2020). Similar systems can also be established in future to enhance the efficiency of HDR in Super Basmati rice.

## Data Availability

All data generated and used is included in this article and supplementary file.

## Notes

**Phenome, Genome & Environment. Chief Editor:** Colleen Doherty
